# Neurodevelopmental and emotional-behavioral outcomes in late-preterm infants: an observational descriptive case study

**DOI:** 10.1186/s12887-018-1293-6

**Published:** 2018-10-08

**Authors:** Roberto Palumbi, Antonia Peschechera, Mariella Margari, Francesco Craig, Arcangelo Cristella, Maria Giuseppina Petruzzelli, Lucia Margari

**Affiliations:** 1 Child Neuropsychiatry Unit, Department of Basic Medical Sciences, Neuroscience and Sense Organs, University of Bari “Aldo Moro”; piazza Giulio Cesare, 1170124 Bari, Italy; 2 Scientific Institute, IRCCS E. Medea, Unit for Severe disabilities in developmental age and young adults, Developmental Neurology and Neurorehabilitation, Brindisi, Italy

**Keywords:** Late preterm, Neurodevelopmental outcome, Emotional-behavioral outcome, Brain development

## Abstract

**Background:**

Over the last decade, several studies investigated the outcomes in children born very preterm. Only recently there has been an increasing interest in the late preterm infants (born between 34 + 0 and 36 + 6 weeks). This population is at high risk of morbidity and mortality in the first years of life. Other studies reported that they are also at risk of long-term developmental problem. Therefore, the aim of this study is to describe the neurodevelopmental and emotional-behavioral outcome in a sample of late preterm patients.

**Methods:**

The study included late preterm children and adolescents who had neuropsychiatric and/or neurological symptoms. They underwent a general, neurocognitive and an emotional-behavioral assessment. Exclusion criteria included: patients affected by Central Nervous System congenital abnormalities, neurodegenerative diseases, genetic disorders, epilepsy, or in pharmacological treatment, or adopted children. A descriptive statistics analysis was performed to describe the sociodemographic and clinical characteristics of patients. Risk factors related to late preterm birth, prevalence of neurodevelopmental disorders, and cognitive functioning were recorded and analyzed.

**Results:**

The sample included 68 LPI (45 males and 23 females) aged from 2 to 16.3 years (mean age 7,5 years), who were affected by one or more neurodevelopmental disorder, including Language Disorder, Attention Deficit Hyperactivity Disorder, Specific Learning Disorder, Developmental Coordination Disorder, Intellectual Disability and Autism Spectrum Disorder. Moreover, in 30.8% of patients, internalizing problems (affective and social skills problem) were detected.

**Conclusions:**

Our results support the importance of a long-term surveillance of late preterm and the great need for more longitudinal large population studies in order to collect data on the neurodevelopmental outcomes of this population.

## Background

Over the last decade, rising rates of the preterm births have been reported; the estimated prevalence is 11.1% of all livebirths worldwide per year [1, 2]. Preterm newborns classifications include the following criteria: Gestational Age, mode of preterm birth (spontaneous versus provider initiated), etiology, or pathophysiological pathways [3]. Infants born between 34 + 0 and 36 + 6 weeks of gestation are classified as “Late Preterm Infants” (LPI); they are the largest group of preterm newborns, accounting for about the 75% of all preterm births. Thus, the burden on public health of this population may be considered reasonable [4]. In the past, many studies focused their attention on the very preterm infants (born at < 32 weeks’ gestation); recently, there has been an increasing interest in the moderate and late preterm. Since they are chronologically close to gestational maturity, until a few years ago, LPI were managed as full-term infants [5].

However, several studies unveiled that LPI are physiologically and metabolically immature and at risk of a major number of hospitalizations in the first years of life. [6–10]. Moreover, recent studies reported that LPI are also at risk of long-term developmental problems, including deficits in neurocognitive/motor domains and behavioral problems. A review article described conflicting results about the impact of late-preterm birth on cognitive functioning, while LPI appeared to develop deficits of school performance and psychiatric disorders in young age and adulthood [11, 12]. A population-based cohort study found that, in late and moderate preterm infants, cognitive impairments were the most common adverse outcome, followed by neuromotor/sensory outcome and neurodevelopmental disability [13]. A recent review analyzing neurodevelopmental outcomes of preterm children reported several results about long-term issues regarding LPI; in fact, this population is characterized by lower cognitive performances and increased risk of special education services support, border-line clinical internalizing and attention problems, and higher risk of psychiatric disorder diagnosis in adulthood [14].

Therefore, to support the importance of a short- and long-term surveillance of this population of infants, the aim of this study is to describe the neurodevelopmental and emotional-behavioral outcomes of a LPI population.

## Methods

### Population

We studied 68 patients late preterm, who were admitted to the Child and Adolescent Neuropsychiatric Unit – University of Bari “Aldo Moro”, between January 2014 and March 2016, for psychopathological or neurological symptoms. We excluded: patients affected by Central Nervous System congenital abnormalities, neurodegenerative diseases, genetic disorders; patients with epilepsy or in psychopharmacological treatment because those factors might have influenced the neurodevelopment and the neurocognitive/emotional-behavioral assessment; adopted children, in case of incomplete or absent data about pre- and perinatal history.

This study was approved by the Local Ethic Committee of Azienda Ospedaliero-Universitaria Policlinico di Bari; all children were recruited after obtaining a written informed consent by their parents; in addition, informed consent was also obtained from the patients who could understand the content and aim of the study.

### General assessment

We collected information related to: parents’ socio-demographic characteristics, family history of neuropsychiatric disorders, obstetric history (previous abortions, multiple pregnancies and risk factors for pregnancy such as utero placental disorders, maternal or fetal diseases) mode of delivery (urgent or elective cesarean section or vaginal delivery) and pre-term delivery causes (medical indications, Premature preterm rupture of the membranes, spontaneous delivery), demographic features of the newborn (gestational age, birth weight) perinatal complications (respiratory complications, jaundice, admission and duration of hospital stay in the neonatal intensive care unit), psychomotor development. All patients underwent physical and neurological examination, laboratory tests (blood cells count, liver and renal functions, metabolic panel).

### Neuropsychiatric assessment

All patients were assessed by highly-trained clinicians, experts in child and adolescent neuropsychiatry. Neuropsychiatric assessment included a neurocognitive and a social-emotional and behavioral evaluation. Cognitive assessment was performed using age-related scales including: Leiter International Performance Scale - Revised (Leiter-R) (for non-verbal patients or in case of language disorder) [15], Wechsler Preschool and Primary Scale of Intelligence – Third edition (WPPSI-III) [16], Wechsler Intelligence Scale for Children – Fourth edition (WISC-IV) [17]. A borderline cognitive functioning is defined by an Intelligence Quotient (IQ) between 70 and 84; a cognitive deficit is defined by an IQ < 70.

The academic achievement was assessed using standardized protocols including: MT Group Reading Tests for Primary School; MT Group Reading Tests for Middle School; MT Group Advanced Reading and Mathematics Tests for the first biennium of Secondary School; Battery for the Evaluation of Developmental Dyslexia and Dysorthography for Primary and Middle school; Evaluation Tests of Calculation Ability for Primary School and Evaluation Tests of Calculation Ability and Problem Solving for Middle School [18–23]. Social-emotional and behavioral problems were assessing using the Child Behaviour Check List (CBCL) [24], a parent-report questionnaire that is composed of several items designed to record, in a standardized format, behavioral problems and competencies of children aged 1.5 through 18 years, as reported by their parents or other primary caregivers. The CBCL allows for the calculation of raw scores and t-scores, normed separately for girls and boys, in 8 different behavioral domains: Withdrawn, Somatic Complaints, Anxious/Depressed, Social Problems, Thought Problems, Attention Problems, Rule-Breaking Behavior and Aggressive Behavior. Neurodevelopment Disorders diagnoses was made according to the Diagnostic and Statistical Manual of Mental Disorders – Fifth edition (DSM-5) criteria [25].

### Statistical analysis

Descriptive statistics were used to describe the sociodemographic and clinicals characteristics of patients. Risk factors related to late preterm birth, prevalence of neurodevelopmental disorders, and cognitive functioning were recorded and analysed. All statistical analysis was conducted using the SPSS software package (version 20.0).

## Results

Socio-demographic features of LPI are described in Table 1. The sample included 68 LPI (45 males and 23 females) aged from 2 to 16.3 years (mean age 7,5 years).

**Table 1:** Socio-demographical characteristics of LP patients

	N (%)
Gender distribution	
Male	45 (66.2%)
Female	23 (33.8%)
Age (mean age ± SD)	
2–16.3 years (7.5 ± 3.4)	
Gestational Age at birth	
34 weeks	19 (27,9)
35 weeks	19 (27,9)
36 weeks	30 (44,2)
Birth weight	
Appropriate for gestational age	37 (54.4)
Low Birth Weight	30 (44.1)
Very Low Birth Weight	1 (1.5)
Extremely Low Birth Weight	–

Late preterm risk factors are summarized in Table 2. Maternal Mean age at the time of the delivery was 31.8 ± 5.2 years. Preterm delivery was performed in 38 patients (55.9%) because of maternal or fetal causes. In 20 patients (29.4%) preterm delivery was spontaneous, and 10 patients (14.7%), were born after a Premature preterm rupture of the membranes. Intrauterine Growth Retardation (IUGR) and oligoidramnios, were the main fetal risk factors of preterm delivery, followed by maternal risk factors, as pre-eclampsia, hypertension and gestational diabetes. The description of the cognitive profile is summarized in Table 3. A Border-line cognitive functioning (Intelligence Quotient = 70–84) was found in 19.2% of patients; an intellectual deficit (Intelligence Quotient < 70) was found in 17.6%.

**Table 2:** Late Preterm birth risk factors

	N (%)
Maternal age	
> 35 years	26 (38,2)
≤ 35 years	42 (61,8)
Twin Pregnancy	12 (17.6)
Neuropsychiatric family History	48 (70,6)
Obstetric Precursors of preterm birth	
Delivery because of maternal or fetal causes	38 (55.9)
Spontaneous Preterm Labor	20 (29.4)
Premature preterm rupture of the membranes	10 (14.7)
Respiratory Complications	10 (14.7)
Jaundice	15 (22.1)
Intensive Neonatal Care	26 (38.2)
Maternal Risk Factors	16 (23.5)
Preeclampsia	9 (13.2)
Hypertension	1 (1.5)
Gestational Diabetes	5 (7.4)
Other Maternal Risk Factors	2 (2.9)
Fetal conditions	20 (29.4)
Intrauterine Growth Retardation	11 (16.2)
Oligoidramnios	6 (8.8)
Other fetal conditions	3 (4.4)

**Table 3:** Cognitive functioning of the sample

	Mean value
Mean Total IQ	91.1
	N (%)
Normal (IQ ≥ 85)	43 (63,2%)
Border-line (IQ = 70–84)	13 (19,2)
Cognitive deficit (IQ < 70)	12 (17,6)

*IQ* Intelligence quotient

Neurodevelopment disorder diagnoses are specified in Table 4: Language Disorder (LD) (32.4%), Attention Deficit Hyperactivity Disorder (ADHD) (23.5%), Specific Learning Disorder (SLD) (22.1%), Developmental Coordination disorder (DCD) (19.1%), Intellectual Disability (ID) (17.6%), Autism Spectrum Disorder (ASD) (13.2%). In 19 out of 68 patients (28%), two disorders were in comorbidity: LD and DCD co-occurred in 13.23%, ASD and ID in 8.8%, ADHD and ASD in 5.9%. The CBCL scores were in a clinical range for internalizing problems (such as affective problems and social skills) in 21 out of 68 LP (30.8%) as reported in Table 5.

**Table 4:** Neurodevelopment disorders diagnoses

	N (%)
Language disorder	22 (32.4)
Attention Deficit Hyperactivity Disorder	16 (23.5)
Specific Learning Disorder	15 (22)
With impairment in reading	8 (11.8)
With impairment in mathematics	4 (5.8)
With impairment in writing	3 (4.4)
Developmental Coordination disorder	13 (19.1)
Intellectual Disability	12 (17.6)
Autism Spectrum Disorder	9 (13.2)

**Table 5:** CBCL scores in clinical range

	N (%)
Internalizing problems	21 (30.8)
Anxious/Depressed	9 (13.2)
Social Problems	8 (11.8)
Somatic Complaints	4 (5.8)

## Discussion

The important increase of late preterm births has been partially related to the greater use of reproductive technologies, and as a result, to a major frequency of maternal-fetal complications during pregnancy [4, 26, 27]. This phenomenon raised a significant interest on the possible outcomes of LPI, including the neurodevelopmental ones. Until a few years ago, LPI were considered as full-term newborns; so, most of the studies were focused on early and/or moderate preterm outcomes. In 2007, Engle et al. defined LPI as a “population at risk”; in fact, as reported also by previous studies, LPI are a group of patients at greater risk of neonatal morbidities and mortality than are term infants (TI). Compared to TI, LPI are more likely to be affected by infections, hypothermia, hypoglycemia, respiratory distress, apnea, jaundice, or feeding difficulties due to labor or delivery complications. Nevertheless, their rehospitalization rates in the first year of life are increased, above all in males and in those who received respiratory support in the primary hospitalization [6]. Besides the neonatal complications and mortality, an increasing number of recent reports suggest that this population is also at increased risk of neurodevelopmental outcomes [10, 13, 28]. Moreover, as emphasized by a recent article review, most of prematurity-related disabilities are described in moderate and late preterm infants, but this group is less studied than those born extremely or very preterm [14].

In our sample, all patients received a neurodevelopmental disorder diagnosis. A Language Disorder was diagnosed in 32.4% of our sample. Previous studies have already found that preterm delivery is considered as a major risk factor for early language impairments [10, 29–31]. A norwegian study compared preterm infants with control term infants in order to verify an association between gestational age and language development outcomes. The results showed an inverse linear relationship between these two variables: the more preterm delivery was early, the more language skills were impaired in the preterm group [32]. A prospective longitudinal cohort study compared moderate-late preterm (MLPT) infants group with healthy full-term controls in order to investigate development outcome of MLPT group at 2 years of age. Results revealed that MLPT children were more likely to have a development delay, more pronounced in the language domains than in the motor ones [33]. In our study, 23.5% of patients were affected by Attention Deficit and Hyperactivity Disorder (ADHD). Previous studies concerning ADHD risk in LPI highlighted controversial results. A retrospective population-based study demonstrated that premature birth is a risk factor for ADHD; results not only showed that the risk declines with the progression of each gestational week, but also that ADHD risk was moderately elevated even in late-preterm and early-term infants [34]. However, on the other hand, a retrospective cohort study found no statistically significant increased risk of developing ADHD for either LPI or early-term infants [31, 35].

In our sample, we diagnosed a Specific Learning Disorder occurred in 22.1% of patients, with an impairment in all academic domains, writing/mathematics and reading. Previous studies already described learning difficulties in LPI. Two reviews found an increased risk for school-related activities in LPI with poor performances in writing/composition, mathematics, speaking/listening and reading [36, 37]. A cohort study evaluated school performance at age 7 in LPI and early preterm infants, revealing that LPI performed worse than full term children in reading and writing [12].

A Developmental Coordination Disorder was found in 19.1% of patients. In literature, is reported that late and moderate preterm children have a higher risk to develop neuro-motor impairment than in term born infants [13, 33].

An Intellectual Disability diagnosis was made in 17.6% of patients. The mean IQ of these patients was 62.3.A recent systematic review investigated long-term cognitive outcomes of late preterm births at ages 2, 4 and 14, did not find significant differences of their intelligent quotient scores with the full-term ones. However, the authors underline that the quality of evidence of the studies examined is poor due to high risk of bias [38].

In 13.2% of patients we found an Autism Spectrum Disorder (ASD) diagnosis. Preterm birth has been already identified as a risk factor for ASD [39, 40]. A large population-based study found that the risk of ASD increased with decreasing gestational age [41]. Another retrospective study identified an elevated probability of ASD among LPI, but the risk relative compared to term infants was not statistically significant [42].

In our sample, 30.8% of patients were in clinical range for internalizing problems (affective problems and social skills) at the CBCL questionnaire. There are only few previous studies reporting emotional-behavior problems in LPI [33, 43, 44]; most of them described a high prevalence of internalizing problems over the externalizing ones.

Therefore, our findings confirmed the evidence that the last half of gestation is a crucial period in the brain development. In fact, at 34 weeks of gestation, brain volume reaches only the 65% of the term brain volume; moreover, the cortical volume in LPI is only 53% of the term volume. In addition, myelination processes show a five-fold increase between 35 and 41 weeks; nevertheless, the gyral and sulcal development goes on until the 40 weeks of gestation. Therefore, LPI brain not only is far from being mature and fully functional, but it is also very vulnerable and susceptible to deprivation of normal environmental influences and to adverse environmental factors [6, 45, 46]. Because of this immaturity, in LPI, the connectivity between brain regions could be disrupted as well; this may explain the occurrence of neurodevelopmental disorders and emotional-behavioral problems in this population [47].

## Conclusions

The rates of neurodevelopmental disorders in our sample are higher than the global prevalence rates in the general population. However, our study has some limitations that need to be considered. First, the size of the sample is quite small. Moreover, we were unable to make comparison with a control group, because of the difficulty in recruiting healthy children and adolescents born full-term. Another limitation is the highly selected group of late preterm children, all of whom were admitted for neuropsychiatric symptoms. In fact, the absence of healthy late preterm children control group prevented the calculation of the prevalence of neurodevelopmental outcomes among children who were born late preterm. Nevertheless, this preliminary study highlights not only the importance of monitoring programs LPI from the first years of life, but also the growing need for more large-population-based and longitudinal studies to identify at an early stage both potential risk factors and first signs of neurodevelopment disorders.

## Abbreviations

ADHD: Attention deficit hyperactivity disorder
ASD: Autism spectrum disorder
CBCL: Child behavior checklist
DSM-5: Diagnostic and statistical manual of mental disorders – Fifth edition
LPI: Late preterm infants
MLPT: Moderate-late preterm
PPROM: Premature preterm rupture of the membranes
TI: Term-infants
WISC-IV: Wechsler intelligence scale for children – Fourth edition
WPPSI-III: Wechsler preschool and primary scale of intelligence – Third edition

## References

[1] Vogel JP, Chawanpaiboon S, Moller AB, Watananirun K, Bonet M, Lumbiganon P. The global epidemiology of preterm birth. Best Pract Res Clin Obstet Gynaecol. 2018;S1521-6934(18)30079–8. 10.1016/j.bpobgyn.2018.04.003. 10.1016/j.bpobgyn.2018.04.003 29779863

[2] Blencowe Hannah, Cousens Simon, Oestergaard Mikkel Z, Chou Doris, Moller Ann-Beth, Narwal Rajesh, Adler Alma, Vera Garcia Claudia, Rohde Sarah, Say Lale, Lawn Joy E (2012). National, regional, and worldwide estimates of preterm birth rates in the year 2010 with time trends since 1990 for selected countries: a systematic analysis and implications. The Lancet.

[3] Kramer Michael S., Papageorghiou Aris, Culhane Jennifer, Bhutta Zulfiqar, Goldenberg Robert L., Gravett Michael, Iams Jay D., Conde-Agudelo Agustin, Waller Sarah, Barros Fernando, Knight Hannah, Villar Jose (2012). Challenges in defining and classifying the preterm birth syndrome. American Journal of Obstetrics and Gynecology.

[4] Teune Margreet J., Bakhuizen Sabine, Gyamfi Bannerman Cynthia, Opmeer Brent C., van Kaam Anton H., van Wassenaer Aleid G., Morris Jonathan M., Mol Ben Willen J. (2011). A systematic review of severe morbidity in infants born late preterm. American Journal of Obstetrics and Gynecology.

[5] Ramachandrappa Ashwin, Jain Lucky (2009). Health Issues of the Late Preterm Infant. Pediatric Clinics of North America.

[6] Engle WA, Tomashek KM, Wallman C, Committee on Fetus and Newborn, American Academy of Pediatrics (2007). “Late-preterm” infants: a population at risk. Pediatrics.

[7] Wang ML, Dorer DJ, Fleming MP, Catlin EA (2004). Clinical outcomes of near-term infants. Pediatrics.

[8] Raju TN, Higgins RD, Stark AR, Leveno KJ (2006). Optimizing care and outcome for late-preterm (near-term) infants: a summary of the workshop sponsored by the National Institute of Child Health and Human Development. Pediatrics.

[9] Shapiro-Mendoza CK, Tomashek KM, Kotelchuck M, Barfield W, Weiss J, Evans S (2006). Risk factors for neonatal morbidity and mortality among “healthy,” late preterm newborns. Semin Perinatol.

[10] Boyle E. M., Poulsen G., Field D. J., Kurinczuk J. J., Wolke D., Alfirevic Z., Quigley M. A. (2012). Effects of gestational age at birth on health outcomes at 3 and 5 years of age: population based cohort study. BMJ.

[11] Cheong Jeanie LY, Doyle Lex W (2012). Increasing rates of prematurity and epidemiology of late preterm birth. Journal of Paediatrics and Child Health.

[12] Chan Evelyn, Quigley Maria A (2014). School performance at age 7 years in late preterm and early term birth: a cohort study. Archives of Disease in Childhood - Fetal and Neonatal Edition.

[13] Johnson Samantha, Evans T Alun, Draper Elizabeth S, Field David J, Manktelow Bradley N, Marlow Neil, Matthews Ruth, Petrou Stavros, Seaton Sarah E, Smith Lucy K, Boyle Elaine M (2015). Neurodevelopmental outcomes following late and moderate prematurity: a population-based cohort study. Archives of Disease in Childhood - Fetal and Neonatal Edition.

[14] Synnes Anne, Hicks Matthew (2018). Neurodevelopmental Outcomes of Preterm Children at School Age and Beyond. Clinics in Perinatology.

[15] Roid GH, Miller LJ (2002). Leiter international performance scale-revised.

[16] Wechsler D (2002). The Wechsler preschool and primary scale of intelligence, third edition (WPPSI-III).

[17] Wechsler D (2012). Wechsler intelligence scale for children. Fourth edition.

[18] Cornoldi C, Colpo G (1998). Prove di lettura MT per la scuola elementare.

[19] Cornoldi C, Colpo G (1995). Nuove prove di lettura MT per la scuola media inferiore.

[20] Cornoldi C, Pra Baldi A, Friso G, Giacomin A, Giofrè D, Zaccaria S (2010). Prove MT Avanzate di Lettura e Matematica 2 per il biennio della scuola superiore di II grado.

[21] Sartori G, Job R, Tressoldi PE (1995). Batteria per la valutazione della dislessia e della disortografia in età evolutiva.

[22] Cornoldi C, Lucangeli D, Bellina M (2002). Test AC-MT. Test di valutazione delle abilità di calcolo. Gruppo MT.

[23] Cornoldi C, Cazzola C (2003). Test di valutazione delle abilità di calcolo e problem solving dagli 11 ai 14 anni.

[24] Achenbach MT. RLA. Manual for the ASEBA school-age forms and profiles. Burlington: University of Vermont; 2001.

[25] American Psychiatric Association (2013). Diagnostic and statistical manual of mental disorder. Fifth Edition.

[26] Joseph KS, Allen AC, Dodds L, Vincer MJ, Armson BA (2001). Causes and consequences of recent increases in preterm birth among twins. Obstet Gynecol.

[27] Hankins GD, Longo M (2006). The role of stillbirth prevention and late preterm (near-term) births. Semin Perinatol.

[28] Poulsen Gry, Wolke Dieter, Kurinczuk Jennifer J, Boyle Elaine M, Field David, Alfirevic Zarko, Quigley Maria A (2013). Gestational Age and Cognitive Ability in Early Childhood: a Population-based Cohort Study. Paediatric and Perinatal Epidemiology.

[29] Wolke Dieter, Samara Muthanna, Bracewell Melanie, Marlow Neil (2008). Specific Language Difficulties and School Achievement in Children Born at 25 Weeks of Gestation or Less. The Journal of Pediatrics.

[30] Guarini A, Sansavini A, Fabbri C, Alessandroni R, Faldella G, Karmiloff-Smith A (2009). Reconsidering the impact of preterm birth on language outcome. Early Hum Dev.

[31] Rabie N Z, Bird T M, Magann E F, Hall R W, McKelvey S S (2015). ADHD and developmental speech/language disorders in late preterm, early term and term infants. Journal of Perinatology.

[32] Zambrana Imac M, Vollrath Margarete E, Sengpiel Verena, Jacobsson Bo, Ystrom Eivind (2015). Preterm delivery and risk for early language delays: a sibling-control cohort study. International Journal of Epidemiology.

[33] Cheong Jeanie L., Doyle Lex W., Burnett Alice C., Lee Katherine J., Walsh Jennifer M., Potter Cody R., Treyvaud Karli, Thompson Deanne K., Olsen Joy E., Anderson Peter J., Spittle Alicia J. (2017). Association Between Moderate and Late Preterm Birth and Neurodevelopment and Social-Emotional Development at Age 2 Years. JAMA Pediatrics.

[34] Sucksdorff M, Lehtonen L, Chudal R, Suominen A, Joelsson P, Gissler M (2015). Preterm birth and poor fetal growth as risk factors of attention-deficit/ hyperactivity disorder. Pediatrics.

[35] Harris M. N., Voigt R. G., Barbaresi W. J., Voge G. A., Killian J. M., Weaver A. L., Colby C. E., Carey W. A., Katusic S. K. (2013). ADHD and Learning Disabilities in Former Late Preterm Infants: A Population-Based Birth Cohort. PEDIATRICS.

[36] de Jong Marjanneke, Verhoeven Marjolein, van Baar Anneloes L. (2012). School outcome, cognitive functioning, and behaviour problems in moderate and late preterm children and adults: A review. Seminars in Fetal and Neonatal Medicine.

[37] McGowan J. E., Alderdice F. A., Holmes V. A., Johnston L. (2011). Early Childhood Development of Late-Preterm Infants: A Systematic Review. PEDIATRICS.

[38] Murray Sarah R., Shenkin Susan D., McIntosh Kirsten, Lim Jane, Grove Benjamin, Pell Jill P., Norman Jane E., Stock Sarah J. (2017). Long term cognitive outcomes of early term (37-38 weeks) and late preterm (34-36 weeks) births: A systematic review. Wellcome Open Research.

[39] Guinchat V, Thorsen P, Laurent C, Cans C, Bodeau N, Cohen D (2012). Pre-peri- and neonatal risk factors for autism. Acta Obstet Gynecol Scand.

[40] Larsson HJ, Eaton WW, Madsen KM, Vestergaard M, Oleson AV, Agerbo E (2005). Risk factors for autism: perinatal factors, parental psychiatric history, and socioeconomic status. Am J Epidemiol.

[41] Kuzniewicz MW, Wi S, Qian Y, Walsh EM, Armstrong MA, Croen LA (2014). Prevalence and neonatal factors associated with autism spectrum disorders in preterm infants. J Pediatr.

[42] Darcy-Mahoney Ashley, Minter Bonnie, Higgins Melinda, Guo Ying, Williams Bryan, Head Zauche Lauren M., Birth Katie (2016). Probability of an Autism Diagnosis by Gestational Age. Newborn and Infant Nursing Reviews.

[43] Potijk Marieke R, de Winter Andrea F, Bos Arend F, Kerstjens Jorien M, Reijneveld Sijmen A (2015). Co-occurrence of developmental and behavioural problems in moderate to late preterm-born children. Archives of Disease in Childhood.

[44] Stene-Larsen K, Lang AM, Landolt MA, Latal B, Vollrath ME (2016). Emotional and behavioral problems in late preterm and early term births: outcomes at child age 36 months. BMC Pediatr.

[45] Walsh Jennifer M., Doyle Lex W., Anderson Peter J., Lee Katherine J., Cheong Jeanie L. Y. (2014). Moderate and Late Preterm Birth: Effect on Brain Size and Maturation at Term-Equivalent Age. Radiology.

[46] Kinney HC (2006). The near-term (late preterm) human brain and risk for periventricular leukomalacia: a review. Semin Perinatol.

[47] Lubsen Julia, Vohr Betty, Myers Eliza, Hampson Michelle, Lacadie Cheryl, Schneider Karen C., Katz Karol H., Constable R. Todd, Ment Laura R. (2011). Microstructural and Functional Connectivity in the Developing Preterm Brain. Seminars in Perinatology.

